# The Pocket Formulary and Synopsis of the British and Foreign Pharmacopœis

**Published:** 1852-01

**Authors:** 


### ARTICLE XI.

*The Pocket Formulary and Synopsis of the British and Foreign Pharmacopœias: Comprising Standard and Approved Formula for the preparations and compounds employed in medical practice.* By HENRY BEASLEY. First American, from the last London edition, corrected, improved and enlarged. Pages 443, 12 mo. Philadelphia: Lindsay & Blakiston, 1852. (From the Publishers through S. C. Griggs & Co.)

This is an exceedingly convenient work for the physician and apothecary. In country practice, such as a large majority of our readers are engaged in, where the practitioner is not only physician, surgeon and accoucheur, but his own apothecary into the bargain, we should think this little work would be very convenient and useful. The title page quoted above, gives a very good idea of the character of the work, hence further comment on our part is unnecessary.

Finding no American editor to this, as well as several other European works lately re-published in this country, we begin to conclude that editing European works is going out of fashion and we very heartily bid the fashion farewell.

